# Unifying dynamics of glass-forming liquids by crystallization temperature

**DOI:** 10.1093/nsr/nwaf423

**Published:** 2025-09-30

**Authors:** Yunhuan Nie, Lijin Wang, Huiming Cao, Pengfei Guan, Ning Xu

**Affiliations:** Hefei National Research Center for Physical Sciences at the Microscale and Department of Physics, University of Science and Technology of China, Hefei 230026, China; School of Physics and Optoelectronic Engineering, Anhui University, Hefei 230601, China; Hefei National Research Center for Physical Sciences at the Microscale and Department of Physics, University of Science and Technology of China, Hefei 230026, China; Advanced Interdisciplinary Science Research (AiR) Center, Ningbo Institute of Materials Technology and Engineering, Chinese Academy of Sciences, Ningbo 315201, China; State Key Laboratory of Advanced Marine Materials, Ningbo Institute of Materials Technology and Engineering, Chinese Academy of Sciences, Ningbo 315201, China; Hefei National Research Center for Physical Sciences at the Microscale and Department of Physics, University of Science and Technology of China, Hefei 230026, China; College of Physics, Guizhou University, Guiyang 550025, China

**Keywords:** glass transition, crystallization temperature, two-step structural relaxation, dynamical heterogeneity, fragility

## Abstract

A crucial question pertaining to the understanding of the elusive glass transition concerns the existence of a universal framework that can describe the dynamics of various glass-forming liquids. Here, we report our achievement of a comprehensive unification of dynamics via the utilization of the crystallization temperature. We provide evidence that the pressure-dependent crystallization temperature links numerous pivotal features of multiple glass-forming liquids, including the super-Arrhenius structural relaxation, dynamical heterogeneity, caging behavior, glass fragility, onset temperature and glass transition temperature. The significant role played by the crystallization temperature reveals the underlying connections between non-equilibrium glass formers and their equilibrium counterparts.

## Teaser

Crystallization temperature provides a universal framework for diverse dynamics of glass-forming liquids.

## INTRODUCTION

Upon fast cooling or compression, virtually all liquids can form glasses, accompanied by a drastic slowdown of dynamics. Although the nature of the glass transition still remains challenging, it is widely recognized that glass-forming liquids exhibit remarkable dynamics that differ significantly from those of simple liquids [1–8]. Specifically, on approaching the glass transition, glass-forming liquids usually undergo a short-time $\beta$ relaxation and a long-time $\alpha$ relaxation. Between the two relaxations, particles are trapped in cages formed by the neighbors, giving rise to a sub-diffusive behavior that manifests as a plateau-like mean squared particle displacement. The plateau value is usually called the Debye–Waller factor [9]. For certain glass-forming liquids, the $\alpha$ (structural) relaxation time $\tau (T)$ shows a super-Arrhenius temperature dependence. Additionally, the particle dynamics exhibits spatiotemporal heterogeneity [8,10–15].

However, beneath these apparent similarities, there appear to be subtle quantitative differences among various glass-forming materials. For example, when the $\alpha$ relaxation time $\tau$ or viscosity $\eta$ is plotted against $T_{\rm g}/T$, i.e., the famous Angell plot, different materials may display varying degrees of deviation from the Arrhenius behavior [1], where $T_{\rm g}$ represents the glass transition temperature corresponding to a given high value of $\tau$ or $\eta$. This deviation defines another important characteristic quantity—the glass fragility $\kappa =\lbrace {{\rm d ln}\tau }/{ {\rm d }(T_{\rm g}/T)} \rbrace |_{_{T=T_{\rm g}}}$. It remains an open question whether different glass formers adhere to the same underlying dynamics, such that their $\tau (T)$ curves can be scaled and superimposed onto a single master curve. Answering this question is pivotal in advancing our understanding of the glass transition.

Some proposals of scaling functions have been made that were claimed to collapse $\tau (T)$ curves of different glass formers under certain conditions [9,16–24]. However, some of these proposals involve parameters that are either abstract or difficult to measure, thereby hampering our comprehension of the underlying mechanisms. Furthermore, beyond the scaling of $\tau (T)$, it remains under debate whether the various characteristics of glass-forming liquids, such as $\alpha$ relaxation, $\beta$ relaxation, dynamical heterogeneity and glass fragility, are interconnected [21,25–31]. Because many of these characteristics are quantified by time-dependent functions, such as the mean squared displacement and the non-Gaussian parameter [3], it is equivalent to inquire whether scaling collapses of these time-dependent functions can be achieved. More ambitiously, can we identify an easily measurable quantity with a clear physical meaning that can achieve not only a scaling collapse of $\tau (T)$ comparable to previous approaches, but also scaling collapses of time-dependent functions for various glass-forming liquids?

For glass formers consisting of particles with varying sizes, we propose the effective crystallization temperature $T_{\rm c}$ as such a quantity. We find that $T_{\rm c}$ is comparable to the onset temperature of glass formers, so the reduced temperature $T/T_{\rm c}$ serves as a measure of the degree of supercooling. At a given $T/T_{\rm c}$, the structural relaxation times of different glass formers with good glass-forming ability are all proportional to the square root of pressure, $\tau \sim p^{-1/2}$. Consequently, we propose a scaling function, which achieves the scaling collapse of the $\tau (T)$ curves of various glass formers across a wide pressure range. For glass formers studied here, the scaling function can be well fitted with the Vogel–Fulcher–Tammann function [3], suggesting that the $\alpha$ relaxation time diverges at $T_{\rm VFT}\approx T_{\rm c}/3$ and the glass fragility is a function of pressure *p*. Meanwhile, when $T/T_{\rm c}$ is fixed, we obtain the collapses for multiple time-dependent functions, including the mean squared particle displacement, intermediate scattering function and non-Gaussian parameter, when time *t* is scaled by $p^{-1/2}$. Our work provides further evidence that equilibrium properties can be used to understand non-equilibrium systems.

## RESULTS

### Parameters and units

Because we will tune the relative softness of the constituent particle species to optimize the glass-forming ability and perform some dimensional analysis using information such as particle size and softness, it is necessary to define the key parameters and units first.

In a system of *N* particles with the same mass *m*, we assign particle *i* ($i=1,2,\dots ,N$) a diameter $\sigma _i$ from a given distribution to fight against crystallization. Assume that particles *i* and *j* interact via the pairwise potential


(1) \begin{eqnarray*}
U(r_{ij}) = \epsilon _{ij} G(r_{ij}),
\end{eqnarray*}

where $r_{ij}$ is the particle separation, $\epsilon _{ij}$ is the characteristic energy scale of the interaction and the functional form of the dimensionless $G(r_{ij})$ is determined by the type of interaction (see the section entitled ‘Materials and methods’ below). If we define


(2) \begin{eqnarray*}
\epsilon _{ij}=\frac{\epsilon _i+\epsilon _j}{2},
\end{eqnarray*}

$\epsilon _i$ ($\epsilon _j$) effectively represents the softness of particle *i* (*j*). By further defining $\epsilon _i=(1+\delta _i)\epsilon$ with $\delta _i\in [-1,1]$ being assigned from a given distribution, we can construct a system of particles with various softness.

Here we show results mainly for binary mixtures of larger (L) and smaller (S) particles. Poly-disperse systems are partially studied to check the generality of the results. For binary mixtures, we let $\sigma _{\rm S}=\sigma$ and $\sigma _{\rm L}=\gamma \sigma$ for S and L particles, respectively, with $\gamma =\sigma _{\rm L}/\sigma _{\rm S}=1.4$ being the diameter ratio. The numbers of L and S particles are $N_{\rm L}$ and $N_{\rm S}=N-N_{\rm L}$. In this work, we set $N_{\rm L}=N_{\rm S}=N/2$. We also set $\delta _{\rm L}=\Delta$ and $\delta _{\rm S}=-\Delta$, so that $\epsilon _{\rm L}=(1+\Delta )\epsilon$ and $\epsilon _{\rm S}=(1-\Delta )\epsilon$. The variation of $\Delta$ makes one species stiffer and the other softer. For poly-disperse systems, particle diameters are from a Gaussian distribution with a mean $\sigma$ and a half height width $\eta \sigma$. Here we use $\eta =0.3$. For particle *i*, we set $\delta _i=\Delta$ if $\sigma _i>\sigma$, and $\delta _i=-\Delta$ otherwise.

For all systems studied here, we set the units of mass, length and energy to be *m*, $\sigma$ and $\epsilon$, respectively. The time and temperature are thus in units of $\sigma m^{1/2}\epsilon ^{-1/2}$ and $\epsilon k_{\rm B}^{-1}$ with $k_{\rm B}$ being the Boltzmann constant. Here we only show results of three-dimensional ($d=3$) systems, where *d* is the dimension of space. We have verified that our results remain valid in two dimensions.

### Effective crystallization temperature and onset temperature

**Figure 1.**
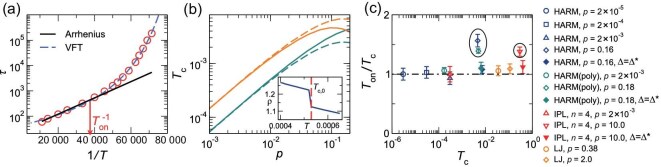
Characteristic temperatures and their relation. (a) Example of the temperature-dependent relaxation time $\tau (T)$ for a binary mixture of HARM particles at $p = 2\times {10}^{-4}$. The solid and dashed lines show the Arrhenius behavior and VFT fitting, respectively. The arrow points to $T_{\rm on}$ below which the Arrhenius behavior is deviated. (b) Examples of the effective crystallization temperatures $T_{\rm c,L}(p)$ (upper) and $T_{\rm c,S}(p)$ (lower) for binary mixtures of HARM particles. The solid and dashed lines are for $\Delta = 0$ and $\Delta =\Delta ^{*}$, respectively. The inset shows the temperature evolution of the density, $\rho (T)$, for mono-disperse HARM systems. The crystallization temperature $T_{\rm c,0}$ is where the density undergoes a discontinuous change. (c) Comparison between the effective crystallization temperature $T_{\rm c}=T_{\rm c,L}$ and onset temperature $T_{\rm on}$ for all the model systems studied in this work. The dash–dot line represents $T_{\rm on} = T_{\rm c}$. We specify $\Delta =\Delta ^{*}$ and poly-disperse systems (‘poly’ in parentheses) in the legend. If not specified, the systems are binary mixtures with $\Delta =0$. Data points showing large deviations from $T_{\rm on}=T_{\rm c}$ are circled. The same systems and legend are presented in Figs 2–4, except for the circled systems whose $T_{\rm on}$ and $T_{\rm c}$ do not match.

In addition to the glass transition temperature, the onset temperature $T_{\rm on}$ is another important characteristic temperature of glass formers [20,32–34]. It separates simple liquids from supercooled liquids, and is hence the crossover from Arrhenius to super-Arrhenius dynamics, as illustrated in Fig. 1a.

To prevent crystallization, we deliberately use particles of varying sizes. If we use identical particles, the system will spontaneously crystallize below a crystallization (or melting) temperature $T_{\rm c,0}$. Under a sufficiently slow cooling rate, there is a discontinuous density change at $T_{\rm c,0}$ when pressure is fixed, as demonstrated in the inset of Fig. 1b. Intuitively, the crystallization temperature $T_{\rm c,0}$ should be irrelevant to glass formers, because particle size variation effectively suppresses crystal formation. Nevertheless, the question arises whether glass formers can still exhibit any vestige of $T_{\rm c,0}$.

Note that, when a crystal-forming liquid is supercooled below $T_{\rm c,0}$, it also exhibits complicated dynamics similar to glass-forming liquids prior to the formation of crystals. In this sense, $T_{\rm c,0}$ of crystal formers could play a similar role as $T_{\rm on}$ of glass formers. Actually, there have been attempts to relate $T_{\rm on}$ to the crystallization (melting) temperature for certain glass formers [34–37], while it has also been shown that their equivalence could be partly coincidental [38]. Furthermore, it has been shown that the crystallization temperature plays important roles in understanding properties of glassy systems including the glass-forming ability and effective temperature [37,39].

The relation between $T_{\rm c,0}$ and $T_{\rm on}$ can be speculated from binary mixtures of L and S particles, which are widely employed as model glass formers. Although the particle size mismatch hinders crystal formation, it is always inevitable to see the formation of micro-phase separation of L and S particles when $T<T_{\rm on}$, as long as the cooling rate is sufficiently slow [39,40]. The micro-phase separation is usually manifested by the coexistence of small crystalline clusters of L or S particles and amorphous mixtures of L and S particles. The formation of crystalline clusters indicates that the temperature of the mixture is below the crystallization temperature of L or S particles forming the clusters. It is therefore reasonable to expect that $T_{\rm on}$ should approximately match the higher crystallization temperature of the two species. The question is how to determine the crystallization temperatures of L and S particles in binary mixtures.

As derived in [39] and in the section entitled ‘Materials and methods’ below, we define *effective crystallization temperatures* experienced by L and S particles in the mixture, denoted $T_{\rm c, L}$ and $T_{\rm c, S}$, respectively, from the simple conversion of units as follows:


(3) \begin{eqnarray*}
T_{\rm c,L}(p) = T_{\rm c,0}\bigg ( \frac{\gamma ^d}{1+\Delta } p \bigg )(1+\Delta ),
\end{eqnarray*}

(4) \begin{eqnarray*}
T_{\rm c,S}(p) = T_{\rm c,0}\bigg ( \frac{1}{1-\Delta } p \bigg )(1-\Delta ).
\end{eqnarray*}

Equations (3) and (4) indicate that $T_{\rm c,L}(p)$ and $T_{\rm c,S}(p)$ can be simply obtained from the $T_{\rm c,0}(p)$ curve by multiplying $T_{\rm c,0}$ and *p*, respectively, by a constant. Note that this simple derivation of effective crystallization temperatures is likely to work only in mixtures with very simple eutectic phase diagrams.

For poly-disperse systems, we can define effective crystallization temperatures for larger ($\sigma _i>\sigma$) and smaller ($\sigma _i<\sigma$) particles in a similar manner to how we define them for binary mixtures, where $\gamma =(2+\eta )/(2-\eta )$ with $\eta$ being the width of the particle diameter distribution, as previously defined. We show that our major findings are applicable to both binary mixtures and poly-disperse systems.

In this work, we study three different types of particle interactions: harmonic (HARM), inverse-power-law (IPL) and Lennard–Jones (LJ) interactions (see their functional forms in the section entitled ‘Materials and methods’ below). Figure 1b illustrates examples of $T_{\rm c,L}(p)$ and $T_{\rm c,S}(p)$ for binary mixtures of HARM particles. The examples are shown for two different values of $\Delta$: 0 and $\Delta ^{*}=(\gamma ^d-1)/(\gamma ^d+1)$.

The $\Delta =0$ HARM systems have been widely used in previous studies. According to Equations (3) and (4), when $\Delta =0$, $T_{\rm c,L}(p)=T_{\rm c,0}(\gamma ^dp)$ and $T_{\rm c,S}(p)=T_{\rm c,0}(p)$, so $T_{\rm c,L}(p)$ can be obtained from $T_{\rm c,0}(p)$ by simply multiplying *p* by a factor of $\gamma ^{-d}$. Figure 1b indicates that $T_{\rm c,L}(p)$ and $T_{\rm c,S}(p)$ (solid lines) intersect at high pressures, because they are non-monotonic at high pressures due to the soft-core nature of the HARM potential. It has been shown that around this high-pressure crossover the glass-forming ability for $\Delta =0$ is weakened, i.e. apparent phase separation of L and S particles occurs [39].

It has been previously suggested that good glass-forming ability of binary mixtures of HARM particles can be recovered at high pressures when $\Delta =\Delta ^{*}$ [39]. In this case, $T_{\rm c,L}(p)=\gamma ^dT_{\rm c,S}(p)$, as shown by the dashed lines in Fig. 1b. It has also been shown that at low pressures $\Delta =0$ and $\Delta =\Delta ^{*}$ lead to comparable glass-forming ability [39]. Here, we are mainly concerned with glass formers that exhibit good glass-forming ability. Consequently, we replace $\Delta =0$ with $\Delta =\Delta ^{*}$ at high pressures when the glass-forming ability for $\Delta =0$ becomes worse.

Now that we have defined effective crystallization temperatures, we can directly compare them with $T_{\rm on}$ to check our previous speculation. Note that, for LJ and IPL potentials with large exponent *n* (see the section entitled ‘Materials and methods’ below), $T_{\rm c,L}(p)$ is always larger than $T_{\rm c,S}(p)$ for both $\Delta =0$ and $\Delta =\Delta ^{*}$, because $T_{\rm c,0}(p)$ increases monotonically. For HARM and IPL potentials with small *n*, $T_{\rm c,L}(p)>T_{\rm c,S}(p)$ when $\Delta =\Delta ^{*}$. However, when $\Delta =0$, strong phase separation occurs when $T_{\rm c,L}(p)<T_{\rm c,S}(p)$ at high pressures [39]. Therefore, for all systems with good glass-forming ability considered here, $T_{\rm c,L}(p)>T_{\rm c,S}(p)$. From our previous speculation, $T_{\rm on}$ should be compared with $T_{\rm c,L}$, which is the higher of the two effective crystallization temperatures. To simplify the notation, we substitute $T_{\rm c,L}$ with $T_{\rm c}$ below, if not specified.

Figure 1c directly compares $T_{\rm c}$ with $T_{\rm on}$ for all three interaction potentials, over a wide range of pressures, and for both binary mixtures and poly-disperse systems. When $\Delta =0$ (open symbols), we observe the expected $T_{\rm on} \approx T_{\rm c}$ relation with some deviations happening for HARM and IPL potentials with $n=4$ at high pressures (circled data points). From our previous analysis, these deviations should be due to the weakening of the glass-forming ability. This is verified by the fact that $T_{\rm on} \approx T_{\rm c}$ is recovered when $\Delta =0$ is replaced with $\Delta =\Delta ^{*}$, as illustrated by the filled symbols in Fig. 1c. Therefore, for systems with good glass-forming ability, $T_{\rm c}$ effectively measures $T_{\rm on}$, and our work provides further direct evidence of the equivalence between the onset temperature of a glass former and the crystallization temperature. Note that it remains unresolved whether this equivalence is generally applicable to all glass formers. Compared to recent studies [34–37], our work extends the equivalence to a wider range of systems with varying interactions and pressures.

In the following, we abandon systems with poor glass-forming ability and only show results for systems with good glass-forming ability and $T_{\rm on}\approx T_{\rm c}$.

### Comprehensive unification of dynamics achieved by effective crystallization temperature

In the study of crystal formation, the nucleation pathways of different crystal-formers are usually compared at the same degree of supercooling characterized by $T/T_{\rm c,0}$ [41–43]. Since we have demonstrated that $T_{\rm on}$ is equivalent to $T_{\rm c}$, it is reasonable to argue that $T/T_{\rm c}$ or $T/T_{\rm on}$ characterizes the degree of supercooling of glass formers. An interesting question then arises: does the degree of supercooling, $T/T_{\rm c}$, govern the dynamics of different glass formers, suggesting that the dynamics follow a much simpler mechanism than those previously proposed [9,16–24]?

**Figure 2.**
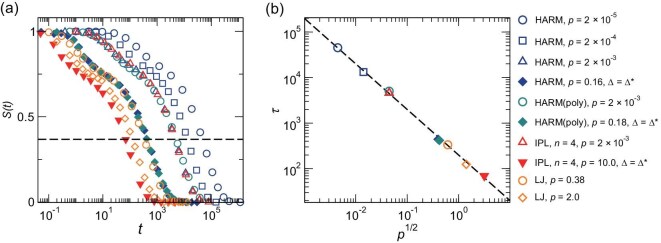
Structural relaxation and relaxation time at the same degree of supercooling $T/T_{\rm c} \approx 0.54$. (a) Intermediate scattering function $S(t)$. The horizontal dashed line shows $S(\tau )=e^{-1}$, at which the relaxation time $\tau$ is determined. (b) Relaxation time $\tau$ versus $p^{1/2}$. The dashed line shows $\tau \sim p^{-1/2}$.

In Fig. 2a, we compare intermediate scattering functions, $S(t)$, of all good glass formers presented in Fig. 1c, approximately at the same $T/T_{\rm c}$. Despite significant differences in their interactions, pressure and particle-size dispersions, the curves exhibit remarkable similarity. It seems that plotting *S* against $t/\tau$, where $\tau$ is the structural relaxation time defined as $S(\tau )=e^{-1}$, would result in the collapse of all the curves onto a single one. We validate this anticipation later. Moreover, Fig. 2b indicates that $\tau \sim p^{-1/2}$ for all systems at the same degree of supercooling, so $p^{-1/2}$ may act as a characteristic time scale at a given degree of supercooling $T/T_{\rm c}$.

In full dimensions, $\tau$ should scale linearly with $(p\sigma /m)^{-1/2}$. Since $\sigma$ and *m* are set to be units of length and mass, respectively, we use the simplified expression $\tau \sim p^{-1/2}$ in reduced units in the following analysis. Note that multiple characteristic time scales can be defined. For example, a characteristic time $t_0\sim \rho ^{-1/3}\sqrt{m/k_{\rm B}T}$ has been proposed within the framework of the IPL potential [44,45], where $\rho$ is the density. The scaling invariance of the IPL potential with exponent *n* implies that $p\sim \rho ^{1+n/3}$ at a given value of $\rho ^{n/3}/T$. Consequently, $t_0\sim \rho ^{-({n}/{6}+{1}/{3})}$ in reduced units. In contrast, our $\tau \sim p^{-1/2}$ scaling yields a characteristic time $\tilde{\tau }\sim \rho ^{-({n}/{6}+{1}/{2})}$. Consequently, the ratio $\tilde{\tau }/t_0\sim \rho ^{-1/6}$. These two time scales differ significantly only when $\rho$ deviates substantially from unity. At present, we are unable to determine which one of $\tilde{\tau }$ and $t_0$ or other alternative time scale is more appropriate for describing IPL systems. However, our $\tilde{\tau }$ appears better than $t_0$ (i.e. $\tilde{\tau }\rho ^{1/6}$ within the IPL framework) to describe dynamics of the various systems studied here (see the online supplementary material).

**Figure 3.**
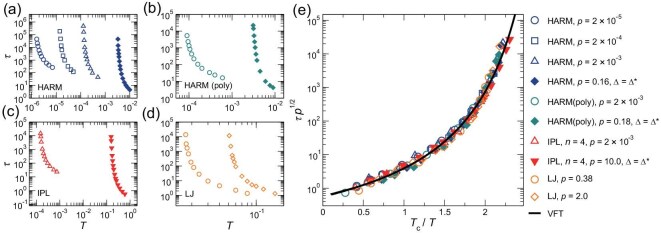
Scaling collapse of the temperature-dependent structural relaxation time. (a–d) Relaxation time $\tau (T)$ for HARM, HARM (poly), IPL and LJ systems measured at different pressures. (e) Scaling collapse of $\tau (T)$ curves when $\tau p^{1/2}$ is plotted against $T_{\rm c}/T$. The black solid curve is the VFT fit of the master curve: $\tau p^{1/2}=0.569 \exp [1.313/(T/T_{\rm c}-0.317)]$.

The results presented in Fig. 2 suggest a functional form for $\tau (T)$:


(5) \begin{eqnarray*}
\tau =p^{-1/2}{\cal F}(T_{\rm c}/T);
\end{eqnarray*}

this may generally describe the super-Arrhenius structural relaxation of all glass formers studied here. Panels (a)–(d) of Fig. 3 show the temperature-dependent relaxation time $\tau (T)$ for all good glass formers presented in Fig. 1c. Interestingly, when we plot $\tau p^{1/2}$ against $T_{\rm c}/T$, all the $\tau (T)$ curves in Fig. 3a–d collapse nicely onto a single master one, as illustrated in Fig. 3e. The scaling collapse indicates that $\tau \sim p^{-1/2}$ holds for all glass formers studied here at any degree of supercooling. The scaling collapse by Equation (5) suggests that the degree of supercooling, $T/T_{\rm c}$, regulates the dynamics of various glass-forming liquids. We present more evidence later.

Note that $\tau \sim p^{-1/2}$ has also been reported for low-pressure HARM systems in previous work, which was shown to break down at high pressures [18]. As discussed above for Fig. 1c, the breakdown should arise from the weakening of the glass-forming ability at high pressures. Crucially, the pivotal role of the degree of supercooling was overlooked by previous studies, which hindered the discovery of Equation (5).

In Fig. S2, we demonstrate that, for the high-pressure systems in Fig. 1c with weakened glass-forming ability, their $\tau (T)$ curves deviate from the master curve in Fig. 3e when Equation (5) is applied. We observe similar deviations when applying other scaling functions proposed in previous studies [9,21]. At the same degree of supercooling, the relaxation time of these poor glass formers is substantially longer than that of good glass formers, due to the pronounced phase separation and crystallization of L and S particles [39].

Now we have achieved the goal of finding a physically simple description of $\tau (T)$ for various good glass formers. In contrast to previous approaches, which relied on physical quantities that are either not easy to measure or physically abstract and implied some complex mechanisms, we realize the scaling collapse of $\tau (T)$ curves solely through the use of $T_{\rm c}(p)$. For example, in [9] and [21], two distinct scaling functions of $\tau (T)$ were proposed, incorporating the Debye–Waller factor and dynamical heterogeneity, respectively. These functions appear to propose two distinct mechanisms for structural relaxation, while the relationship between the Debye–Waller factor and dynamical heterogeneity remains unclear. Apparently, our approach reveals a much simpler underlying mechanism: the structural relaxation is primarily governed by the degree of supercooling. Furthermore, our approach suggests that the Debye–Waller factor and dynamical heterogeneity are indeed related, with $T/T_{\rm c}$ serving as the link. We provide evidence to support this connection later.

There exist multiple functional forms of the scaling function in Equation (5), each based on a different theoretical perspective of the glass transition [2,3,22,23,46–49]. In Fig. S1, we fit the master curve in Fig. 3e with various functions. The Vogel–Fulcher–Tammann (VFT) function is one of the best to fit all the data over the whole range of temperatures:


(6) \begin{eqnarray*}
\tau =\tilde{\tau }{\rm exp}\bigg (\frac{A}{T-T_{\rm VFT}}\bigg )=ap^{-1/2}{\rm exp}\bigg (\frac{bT_{\rm c}}{T-cT_{\rm c}}\bigg ),
\end{eqnarray*}

with *a, b* and *c* fitting parameters. Our fitting (solid line in Fig. 3e) gives $a\approx 0.569$, $b\approx 1.313$ and $c\approx 0.317$, which are independent of the systems. We can draw two conclusions from Equation (6).

Firstly, $T_{\rm VFT}=cT_c$ is often considered as the glass transition temperature at which the relaxation time $\tau$ is supposed to diverge within the VFT framework. In our case, we have $T_{\rm VFT}\approx 0.317 T_{\rm c}$, indicating that the glass transition temperature is linearly related to the effective crystallization temperature. We can now establish a relation between $T_{\rm VFT}$ and $T_{\rm on}$ as follows: $T_{\rm VFT}\approx 0.317 T_{\rm c}\approx T_{\rm on}/3$. This is in good agreement with the results of some other studies [50–53].

Secondly, $A/T_{\rm VFT}=b/c$ is commonly used as an indicator of glass fragility. Now we observe that, as long as the scaling collapse is valid, the fragility indicator remains constant for various glass formers. On the other hand, if we directly calculate the aforementioned fragility $\kappa =\lbrace {{\rm d ln}\tau }/{ {\rm d}(T_{\rm g}/T)} \rbrace |_{_{T=T_{\rm g}}}$, using Equation (5), we have


(7) \begin{eqnarray*}
\kappa =\xi \frac{{\cal F}^{\prime }(\xi )}{{\cal F}(\xi )},
\end{eqnarray*}

where $\xi =T_{\rm c}/T_{\rm g}$. Here, $T_{\rm g}$ is the experimental glass transition temperature at which $\tau =\tau _{\rm g}$. Note that the $\kappa$ values of different glass formers are usually compared at the same $\tau _{\rm g}$. Equation (5) indicates that $\tau _{\rm g}p^{1/2}={\cal F}(\xi )={\cal F}(T_{\rm c}/T_{\rm g})$. Consequently, Equation (7) demonstrates that $\kappa$ can also be written as a function of $\tau _{\rm g}p^{1/2}$. At a given $\tau _{\rm g}$, $\kappa$ is just a function of *p*. Therefore, how the fragility $\kappa$ varies with pressure *p* is determined by the functional form of ${\cal F}(\xi (p))$. Since the VFT form can fit our data well, using Equation (6) can provide us with the pressure dependence of $\kappa$,


(8) \begin{eqnarray*}
\kappa =\frac{b\xi }{(1-c\xi )^2}=\frac{c}{b}\bigg ( {\rm ln} \frac{\tau _{\rm g}p^{1/2}}{a} \bigg )^2 + {\rm ln} \frac{\tau _{\rm g}p^{1/2}}{a}.
\end{eqnarray*}

Apparently, the fragility increases monotonically with increasing $\xi$ or pressure *p*. The same pressure dependence of the fragility was reported in previous studies of HARM systems [54]. Here, we find that this conclusion also applies to different systems, regardless of their interaction potentials.

The VFT function implies the divergence of $\tau$ at a finite temperature $T_{\rm VFT}$. In contrast, Elmatad *et al.* [22,23] proposed a parabolic (ECG) function that predicts the divergence of $\tau$ only at $T=0$. It has been shown that the ECG function can fit $\tau (T)$ down to the glass transition temperature, where the VFT function may fail [23]. As shown in Fig. S1, the ECG function can also fit our low $T/T_{\rm c}$ data well. While the VFT and ECG functions support and oppose, respectively, the existence of a thermodynamic glass transition at finite temperatures—an issue that remains unsolved—within the ECG framework, Equation (7) still predicts that $\kappa$ increases with increasing *p* or $\xi$.

**Figure 4.**
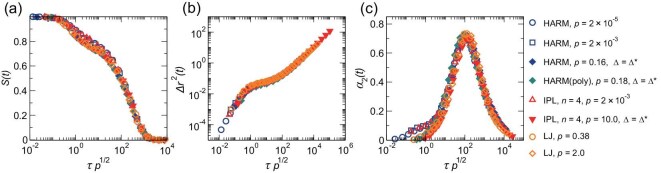
Scaling collapse of multiple time-dependent functions at the same degree of supercooling $T/T_{\rm c} \approx 0.54$. (a) Intermediate scattering function $S(t)$, (b) mean squared displacement $\Delta r^{2}(t)$ and (c) non-Gaussian parameter $\alpha _{2}(t)$ versus $t p^{1/2}$. The symbols employed are consistent with those depicted in Fig. 1.

As discussed previously for Fig. 2, at a given degree of supercooling $T/T_{\rm c}$, the intermediate scattering function curves of different systems may collapse when they are plotted against $\tau p^{1/2}$. This is confirmed by Fig. 4a. More surprisingly, panels (b)–(c) of Fig. 4 demonstrate that the mean squared displacement $\Delta r^2(t)$ and non-Gaussian parameter $\alpha _2(t)$ also exhibit scaling collapse when plotted against $tp^{1/2}$. The scaling collapse observed in the time-dependent functions suggests that the characteristics of glass-forming liquids, including the $\alpha$ relaxation, $\beta$ relaxation, dynamical heterogeneity and Debye–Waller factor, are all linked to $T_{\rm c}(p)$, thus indicating their interconnection. Benefiting from the use of the degree of supercooling, we are able to achieve a comprehensive unification of dynamics of various glass-forming liquids.

## DISCUSSION

In this work, we study dynamics of several simple glass formers consisting of spherical particles over a wide range of pressures. By defining the effective crystallization temperatures of glass formers and finding the equivalence between the onset temperature and the higher effective temperature, we define the degree of supercooling and unify the dynamics of various glass formers. Our unification includes the scaling collapses of the temperature-dependent structural relaxation time and characteristic time-dependent functions. Our approach suggests that the glass transition temperature is linearly related to the effective crystallization temperature $T_{\rm c}$ and that the glass fragility increases with increasing pressure.

The unification of dynamics reported here links multiple widely concerned characteristics of glass-forming liquids, including the super-Arrhenius structural relaxation, dynamical heterogeneity and caging. The interconnection between these characteristics has been a sought-after understanding in supercooled liquids. The cornerstone of our achievement lies in the definition and utilization of the degree of supercooling. It is intriguing that the equilibrium crystallization temperature plays such a pivotal role in the dynamics of non-equilibrium glass-forming liquids, suggesting underlying connections between non-equilibrium systems and their equilibrium counterparts [37,39].

Our main results are derived from systems with repulsion-dominated interactions and good glass-forming ability. We demonstrate that the weakening of the glass-forming ability can lead to deviations from our scaling. Other factors, such as attractive interactions and material complexity, may also influence the scaling, which require further investigations.

## MATERIALS AND METHODS

### System details

In this work, we consider three-dimensional systems of $N=1000$ particles in a cubic box with a side length *L*. Periodic boundary conditions are applied in all directions. We have partly verified that our results do not show strong system size dependence. For HARM, LJ and IPL potentials, the dimensionless functions $G(r_{ij})$ in Equation (1) are


(9) \begin{eqnarray*}
G(r_{ij}) = \frac{1}{2} \bigg (1-\frac{r_{ij}}{\sigma _{ij}}\bigg )^{2} \Theta \bigg (1-\frac{r_{ij}}{\sigma _{ij}}\bigg ),
\end{eqnarray*}

(10) \begin{eqnarray*}
G(r_{ij}) &=& \frac{1}{72} \bigg [ \bigg ( \frac{\sigma _{ij}}{r_{ij}} \bigg )^{12} -\bigg ( \frac{\sigma _{ij}}{r_{ij}} \bigg )^{6} \bigg ] \\
&&\quad +\, \chi _{\rm LJ}(r_{ij}),
\end{eqnarray*}

(11) \begin{eqnarray*}
G(r_{ij}) = \bigg ( \frac{\sigma _{ij}}{r_{ij}} \bigg )^{n} + \chi _{\rm IPL}(r_{ij}),
\end{eqnarray*}

respectively, where $\sigma _{ij}$ is the sum of the radii of particles *i* and *j*, and $\Theta (x)$ is the Heaviside step function. For LJ and IPL potentials, we set a potential cutoff at $r_{ij}=2.5\sigma _{ij}$ and $\sigma _{ij}$, respectively. The terms $\chi _{\rm LJ}$ and $\chi _{\rm IPL}$ in Equations (10) and (11) are to guarantee that *G* and $G^{\prime }$ vanish at the potential cutoff.

### Derivation of effective crystallization temperatures

For mono-disperse crystal formers with particle diameter $\sigma$ and characteristic energy scale (softness) of the interaction $\epsilon$, the crystallization temperature $T_{\rm c,0}$ and pressure *p* are in units of $\epsilon k_{\rm B}^{-1}$ and $\epsilon \sigma ^{-d}$, respectively. For a binary mixture at a pressure $p\times \epsilon \sigma ^{-d}$, L and S particles could experience different crystallization temperatures due to their differences in particle size and softness. We denote $P_{\rm L}$ and $P_{\rm S}$ as the *effective pressures* of L and S particles in their own units, respectively. Because L and S particles actually have the same pressure *p* in units of $\epsilon \sigma ^{-d}$, the conversion of units leads to


(12) \begin{eqnarray*}
P_{\rm L}\times \epsilon _{\rm L}\sigma _{\rm L}^{-d} &=& P_{\rm L}\times \epsilon (1+\Delta )(\gamma \sigma )^{-d} \\
&=& p\times \epsilon \sigma ^{-d},
\end{eqnarray*}

(13) \begin{eqnarray*}
P_{\rm S}\times \epsilon _{\rm S}\sigma _{\rm S}^{-d} &=& P_{\rm S}\times \epsilon (1-\Delta )\sigma ^{-d} \\
&=& p\times \epsilon \sigma ^{-d},
\end{eqnarray*}

from which we have


(14) \begin{eqnarray*}
P_{\rm L} = \frac{\gamma ^d}{1+\Delta } p,
\end{eqnarray*}

(15) \begin{eqnarray*}
P_{\rm S} = \frac{1}{1-\Delta } p.
\end{eqnarray*}

Similarly, we can perform the conversion of units for *effective crystallization temperatures*  $T_{\rm c, L}(p)$ and $T_{\rm c, S}(p)$:


(16) \begin{eqnarray*}
T_{\rm c,L}(p)\times \epsilon k_{\rm B}^{-1} = T_{\rm c,0}\left( P_{\rm L} \right)\times \epsilon _{\rm L}k_{\rm B}^{-1},
\end{eqnarray*}

(17) \begin{eqnarray*}
T_{\rm c,S}(p)\times \epsilon k_{\rm B}^{-1} = T_{\rm c,0}\left( P_{\rm S} \right)\times \epsilon _{\rm S}k_{\rm B}^{-1}.
\end{eqnarray*}

Then, we finally have expressions for $T_{\rm c, L}(p)$ and $T_{\rm c, S}(p)$ as described in Equations (3) and (4), respectively.

### Characteristic quantities of supercooled liquids

We perform molecular dynamics simulations at constant $NpT$. For mono-disperse systems, we calculate the time-averaged density as a function of temperature. For glass-forming liquids, we calculate the self-part of the intermediate scattering function for larger particles:


(18) \begin{eqnarray*}
S(t)=\frac{1}{N_{\rm L}} \sum _{j}\exp ({\rm i} \vec{q} \cdot [\vec{r}_{j}(t)-\vec{r}_{j}(0)]).
\end{eqnarray*}

Here the sum is over all larger particles (L particles for binary mixtures and particles with diameters larger than $\sigma$ for poly-disperse systems), $\vec{r}_j(t)$ is the position of particle *j* at time *t*, and $\vec{q}$ is chosen in the *x* direction with $q=|\vec{q}|$ satisfying the periodic boundary conditions and being approximately the value at the first peak of the static structure factor. To locate the onset temperature, we fit the intermediate scattering function $S(t)$ with the Kohlrausch–Williams–Watts stretched exponential form, $S(t) \sim \exp [-(t/\tau )^\beta ]$. For simple liquids with Arrhenius behavior, $\beta = 1$. When $T < T_{\rm on}$ and the liquids exhibit super-Arrhenius behavior, $\beta < 1$. We set $T_{\rm on}$ as the temperature at which $\beta$ decays to 0.8 with the variation being evaluated from 0.75 to 0.85 [55,56]. The mean squared displacement $\Delta {r^2(t)}$ and the non-Gaussian parameter $\alpha _2(t)$ for larger particles are calculated as


(19) \begin{eqnarray*}
\Delta {r^2(t)} = \langle [ \Delta \vec{r}(t)] ^2 \rangle ,
\end{eqnarray*}

(20) \begin{eqnarray*}
\alpha _2(t) = \frac{ \langle [\Delta \vec{r}(t)]^4 \rangle }{(1+ 2 / d) \langle [\Delta \vec{r}(t)]^2 \rangle ^2} - 1,
\end{eqnarray*}

where $\Delta \vec{r}(t)$ is the particle displacement over a time duration *t, d* is the dimension of space and $\langle \cdot \rangle$ denotes the average over particles and configurations.

## Supplementary Material

nwaf423_Supplemental_File
