# Supplementary material for: Unifying dynamics of glass-forming liquids by crystallization temperature
Source: Natl Sci Rev. 2025 Sep 30;12(12):nwaf423. doi: 10.1093/nsr/nwaf423 (PMC12636646; doi:10.1093/nsr/nwaf423)
Supplement: nwaf423_Supplemental_File [file nwaf423_supplemental_file.pdf]

Supplementary Material: Unifying dynamics of glass-forming liquids by crystallization temperature

Contents

1. Values of characteristic quantities 1

2. Fitting functions 2

3. Temperature-dependence of structural relaxation time for poor glass-forming liquids 2

4. Scaling collapse of Intermediate scattering functions for small particles 3

5. Scaling collapse of $\tau(T)$ curves using the $\tau \sim p^{-1/2}\rho^{1/6}$ scaling 3

References 4

1. Values of characteristic quantities

TABLE S1: Pressure and temperature values in reduced units used in this work.

Potential Model	pressure	$T_{\mathrm{on}} \pm \delta T_{\mathrm{on}}$	$T_{\mathrm{c}} \pm \delta T_{\mathrm{c}}$
**HARM**	2×10^{-5}	$(3.0 \pm 0.5) \times 10^{-6}$	$(3.0 \pm 0.2) \times 10^{-6}$
**HARM**	2×10^{-4}	$(3.1 \pm 0.5) \times 10^{-5}$	$(3.0 \pm 0.2) \times 10^{-5}$
**HARM**	2×10^{-3}	$(2.8 \pm 0.5) \times 10^{-4}$	$(3.0 \pm 0.2) \times 10^{-4}$
**HARM**	0.16	$(7.2 \pm 0.8) \times 10^{-3}$	$(4.6 \pm 0.2) \times 10^{-3}$
**HARM**	$0.16(\Delta = \Delta^{*})$	$(7.8 \pm 0.8) \times 10^{-3}$	$(7.2 \pm 0.3) \times 10^{-3}$
**HARM (poly)**	2×10^{-3}	$(1.8 \pm 0.3) \times 10^{-4}$	$(1.7 \pm 0.2) \times 10^{-4}$
**HARM (poly)**	0.18	$(6.8 \pm 0.6) \times 10^{-3}$	$(4.9 \pm 0.3) \times 10^{-3}$
**HARM (poly)**	$0.18(\Delta = \Delta^{*})$	$(7.4 \pm 1.0) \times 10^{-3}$	$(6.4 \pm 0.3) \times 10^{-3}$
**IPL(n=4)**	2×10^{-3}	$(3.0 \pm 0.6) \times 10^{-4}$	$(3.0 \pm 0.2) \times 10^{-4}$
**IPL(n=4)**	10.0	0.4 ± 0.05	0.29 ± 0.02
**IPL(n=4)**	$10.0(\Delta = \Delta^{*})$	0.44 ± 0.06	0.39 ± 0.02
**LJ**	0.38	0.037 ± 0.004	0.035 ± 0.001
**LJ**	2.0	0.12 ± 0.02	0.11 ± 0.01

2. Fitting functions

Figure S1 compares multiple functional forms proposed to describe the temperature-dependence of the relaxation time, $\tau(T)$. Here we compare the Vogel-Fulcher-Tammann (VFT) function with other functions, i.e., Elmatad-Chandler-Garrahan (ECG), Avramov-Milchev (AM), Mauro-Yue-Ellison-Gupta-Allan (MYEGA), and mode coupling(MC) equations [1–6]. All functions provide good fits to the low-temperature data. The VFT function appears one of the best that fits the data over the whole temperature range.

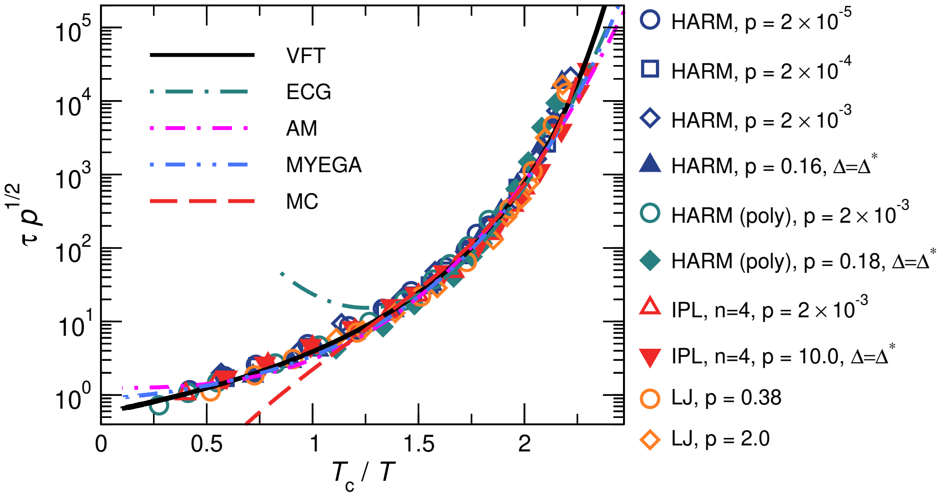

FIG. S1: Comparison of different functional forms in describing the temperature-dependent relaxation time. These functions are $y = 0.569\exp[1.313/(x-0.317)]$ (VFT), $y = 15.36\exp[6.77(x^{-1}-1.252)^2]$ (ECG), $y = 1.248\exp[(0.962/x)^{2.849}]$ (AM), $y = 0.872\exp[(0.548/x)\exp(0.908/x)]$ (MYEGA), and $y = 0.449(x-0.419)^{-2.946}$ (MC), where $x = T/T_c$ and $y = \tau p^{1/2}$. If not specified in the legend, the systems are binary mixtures with $\Delta = 0$.

3. Temperature-dependence of structural relaxation time for poor glass-forming liquids

In Fig. S2, we add the $\tau(T)$ curves of the three poor glass-forming liquids in Fig. 1(c) of the main text to Fig. 3(e) of the main text. They deviate significantly from the master curve due to their weakened glass-forming ability and pronounced phase separation of large and small particles.

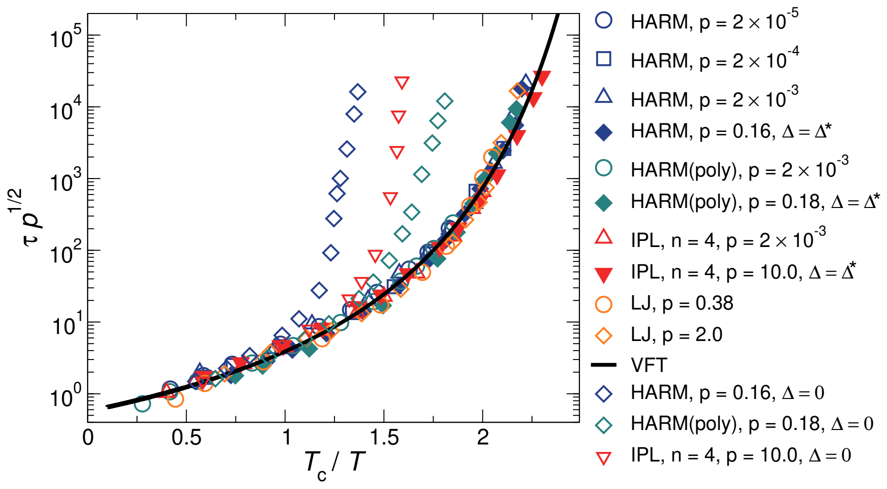

FIG. S2: Comparison of the $\tau(T)$ curves of poor glass-forming liquids with those of good glass-forming liquids shown in Fig.3(e) of the main text. If not specified in the legend, the systems are binary mixtures with $\Delta = 0$.

4. Scaling collapse of Intermediate scattering functions for small particles

In Fig. 4(a) of the main text, we show that intermediate scattering functions $S(t)$ for large particles can collapse nicely when S is plotted against $tp^{1/2}$. In Fig. S3, we demonstrate that this is also the case for small particles.

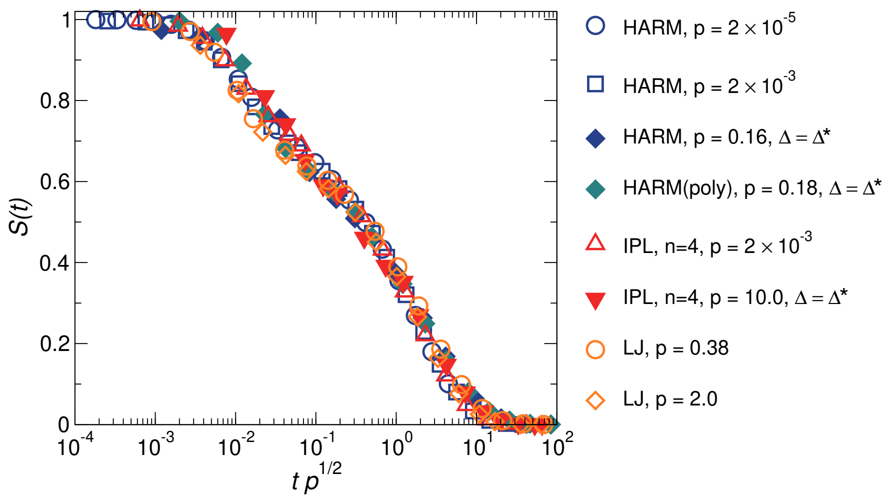

FIG. S3: Scaling collapse of Intermediate scattering function $S(t)$ for small particles at the same degree of supercooling $T/T_c \approx 0.54$. If not specified in the legend, the systems are binary mixtures with $\Delta = 0$.

5. Scaling collapse of $\tau(T)$ curves using the $\tau \sim p^{-1/2}\rho^{1/6}$ scaling

As discussed in the main text, within the inverse-power-law (IPL) potential framework, the characteristic time $\tilde{\tau} \sim p^{-1/2}$ should be proportional to $t_0\rho^{-1/6}$, where ρ is the density, and t_0 is the characteristic time proposed previously for the IPL systems [7], as defined in the main text. To check whether t_0 can be used to collapse our $\tau(T)$ curves shown in Fig. 3(e) of the main text, we plot $\tau p^{1/2}\rho^{-1/6} \sim \tau t_0^{-1}$ against T_c/T in Fig. S4. The scaling collapse appears worse than that in Fig. 3(e) of the main text.

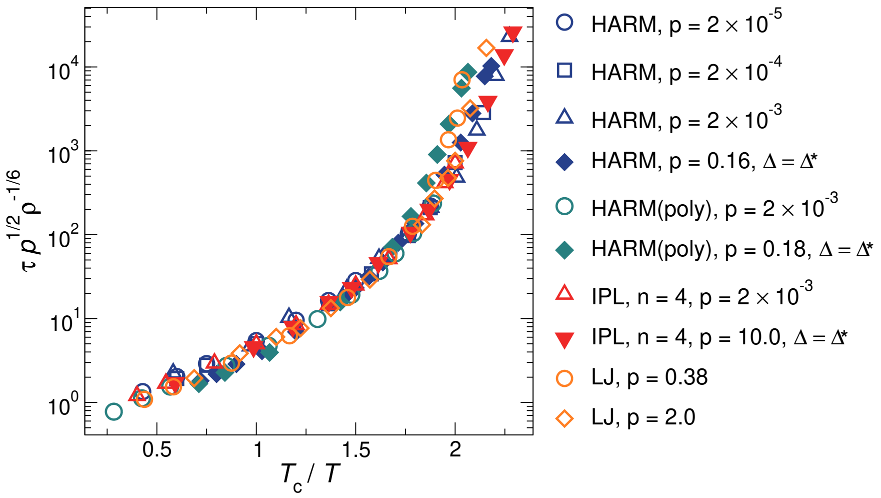

FIG. S4: Scaling collapse of $\tau(T)$ curves when $\tau p^{1/2}\rho^{-1/6}$ is plotted against T_c/T. If not specified in the legend, the systems are binary mixtures with $\Delta = 0$.

---

[1] Debenedetti PG and Stillinger FH. Supercooled liquids and the glass transition. *Nature* 2001; **410**, 259-267.
[2] Tanaka H, Kawasaki T, Shintani H and Watanabe K. Critical-like behaviour of glass-forming liquids. *Nat. Mater.* 2010; **9**, 324.
[3] Berthier L and Biroli G. Theoretical perspective on the glass transition and amorphous materials. *Rev. Mod. Phys.* 2011; **83**, 587.
[4] Elmatad YS, Chandler D and Garrahan JP. Corresponding states of structural glass formers. *J. Phys. Chem. B* 2009; **113**, 5563.
[5] Avramov I and Milchev A. Effect of disorder on diffusion and viscosity in condensed systems. *J. Non-Cryst. Solids* 1998; **104**, 253; Averamov I. Viscosity of glass forming melts. *J. Non-Cryst. Solids* 1998; **238**, 6.
[6] Mauro JC, Yue Y, Ellison AJ, Gupta PK and Allan DC. Viscosity of glass-forming liquids. *Proc. Natl. Acad. Sci. U.S.A.* 2009; **106**, 19780.
[7] Schrøder TB, Bailey NP, Pedersen UR, Gnan N, Dyre JC. Pressure-energy correlations in liquids. III. Statistical mechanics and thermodynamics of liquids with hidden scale invariance. *J. Chem. Phys.* 2009; **131** 234503.
